# HIF-1α promoted vasculogenic mimicry formation in lung adenocarcinoma through NRP1 upregulation in the hypoxic tumor microenvironment

**DOI:** 10.1038/s41419-021-03682-z

**Published:** 2021-04-13

**Authors:** Ran Fu, Wenwen Du, Zongli Ding, Yi Wang, Yue Li, Jianjie Zhu, Yuanyuan Zeng, Yulong Zheng, Zeyi Liu, Jian-an Huang

**Affiliations:** 1 Department of Pulmonary and Critical Care Medicine, The First Affiliated Hospital of Soochow University, Suzhou, 215006 China; 2 Suzhou Key Laboratory for Respiratory Diseases, Suzhou, 215006 China; 3 Department of Respiratory Medicine, The Affiliated Huai’an Hospital of Xuzhou Medical University, Huai’an, 223002 China; 4 Institute of Respiratory Diseases, Soochow University, Suzhou, 215006 China

**Keywords:** Lung cancer, Cell migration

## Abstract

Neovascularization is a key factor that contributes to tumor metastasis, and vasculogenic mimicry (VM) is an important form of neovascularization found in highly invasive tumors, including lung cancer. Despite the increasing number of studies focusing on VM, the mechanisms underlying VM formation remain unclear. Herein, our study explored the role of the HIF-1α/NRP1 axis in mediating lung adenocarcinoma metastasis and VM formation. HIF-1α, NRP1 expression, and VM in lung adenocarcinoma (LUAD) patient samples were examined by immunohistochemical staining. Quantitative real-time (qRT-PCR), western blot, transwell assay, wound healing assay, and tube formation assay were performed to verify the role of HIF-1α/NRP1 axis in LUAD metastasis and VM formation. ChIP and luciferase reporter assay were used to confirm whether NRP1 is a direct target of HIF-1α. In LUAD tissues, we confirmed a positive relationship between HIF-1α and NRP1 expression. Importantly, high HIF-1α and NRP1 expression and the presence of VM were correlated with poor prognosis. We also found that HIF-1α could induce LUAD cell migration, invasion, and VM formation by regulating NRP1. Moreover, we demonstrated that HIF-1α can directly bind to the NRP1 promoter located between −2009 and −2017 of the promoter. Mechanistically, MMP2, VE-cadherin, and Vimentin expression were affected. HIF-1α plays an important role in inducing lung adenocarcinoma cell metastasis and VM formation via upregulation of NRP1. This study highlights the potential therapeutic value of targeting NRP1 for suppressing lung adenocarcinoma metastasis and progression.

## Background

Non-small cell lung cancer (NSCLC) is the leading cause of cancer-related deaths worldwide. And it also accounts for 30% of all lung cancer cases^1^. NSCLC features uncontrollable mortality and invasiveness, high recurrence rates, and strong angiogenesis. Tumor angiogenesis is an independent prognostic factor in cancer and is associated with widespread hypoxia in tumors and a relatively inadequate blood supply^2^. Moreover, accumulating studies have also demonstrated a new method of angiogenesis under hypoxic conditions named vasculogenic mimicry (VM). VM is characterized by vascular-like structures and results in the formation of a new blood supply system in aggressive tumor cells, which is positive for periodic acid-Schiff (PAS) staining^3,4^. VM has been found in various malignant tumors, including NSCLC, indicating more aggressive tumor behavior^5^.

Within the tumor microenvironment, hypoxia is the most common phenomenon because of the vast energy and oxygen consumption of tumor cells. Hypoxia, which is orchestrated by hypoxia-inducible factor-1α (HIF-1α), contributes to tumor metabolism, angiogenesis, and cell survival^6,7^. It has been well established that the transcription factor HIF-1α enhances tumor cell motility and invasiveness and contributes to the epithelial–mesenchymal transition (EMT) process, which is crucial for VM formation^8,9^. Overexpression of HIF-1α in primary gallbladder carcinoma is related to vasculogenic mimicry and unfavorable prognosis^10^. In gastric adenocarcinoma, STAT3, p-STAT3, and HIF-1α are associated with vasculogenic mimicry and impact patient survival^11^. Moreover, it has been reported that HIF-1α promotes vasculogenic mimicry formation in hepatocellular carcinoma through upregulated LOXL2 in the hypoxic tumor microenvironment^12^.

NRP1 is a transmembrane glycoprotein that acts as a coreceptor for numerous extracellular ligands, including transforming growth factor-beta (TGF-β), vascular endothelial growth factor (VEGF), and platelet-derived growth factor (PDGF)^13,14^. NRP1 is considered to be an important driver of malignant tumor progression. NRP1 is overexpressed in a variety of tumor tissues and is involved in the development of axon guidance and remyelination, immune response, angiogenesis, cell survival, migration, and invasion^15,16^. It has been reported that the NRP1^+^ cell subpopulation showed an increased expression of the pluripotency markers OCT-4, Bmi-1, and NANOG as well as higher cell migratory, clonogenic, and self-renewal capacities^17^. Our previous published study pointed out that NRP1 contributed to TGF-β1-induced EMT and metastasis in NSCLC by binding with TGFβRII^18^. In addition, we demonstrated that NRP1 promotes NSCLC metastasis via EGFR signaling^19^. The role of NRP1 in the hypoxic tumor microenvironment in mediating VM has remained largely unknown.

In our present study, we demonstrate that NRP1 is the direct target of HIF-1α that promotes VE-cadherin, MMP2, and Vimentin expression under hypoxic conditions. HIF-1α plays an important role in the development of VM in lung cancer by upregulating NRP1, indicating the potential therapeutic value of targeting NRP1 for the suppression of NSCLC metastasis and progression.

## Materials and methods

### Patients and samples

A total of 169 primary lung adenocarcinoma specimens were obtained from the Affiliated Huai’an Hospital of Xuzhou Medical University. All participants were provided with written informed consent at the time of recruitment. The diagnosis of lung adenocarcinoma was verified for all of these samples based on their histological and pathological characteristics. Before tissue sampling, none of the patients had received any therapy, such as chemotherapy or radiotherapy. The present study was approved by the Ethics Committee of the Affiliated Huai’an Hospital of Xuzhou Medical University. All samples were stored frozen at −80 °C.

### Immunohistochemistry and analysis

The detailed procedures were performed as previously described^20^. The antibodies used in the current study were as follows: anti-HIF-1α (D1S7W) and anti-NRP1 (A-12, Santa Cruz Biotechnology, CA, USA). In addition, we also performed CD34/PAS double staining to detect VM expression in tumor tissues. CD34 staining (GB121693, Servicebio, Wuhan, China) was performed on the sections prior to PAS staining (GP1039, Servicebio, Wuhan, China). Then, all slides were treated with periodic acid solution for 10 min and submerged in Schiff solution for 15 min at 37 °C. After washing, all sections were counterstained with hematoxylin, dehydrated, and mounted. And we applied the quantitative scoring methods to evaluate the staining results. The total score was calculated by multiplying the percentage of positive cells (P) by the intensity (I). Formula: *Q* = *P* × *I*; *P*-score was assigned that represented the estimated proportion of antibody-stained tumor cells. For positive cells, 0 (<5%), 1 (5–25%), 2 (26–50%), or 3 (51–75%), 4 (76–100%) means the percentage of malignant cells staining within carcinomatous areas. *I*-score was assigned that estimated the average staining intensity of positive tumor cells. For intensity, 0 means no staining, 1 means weak staining, 2 means moderate staining, and 3 means strong staining. Finally the R language was applied to draw the correlation analysis.

### Cell culture and CoCl_2_ treatment

The A549 and SPC-A1 lung adenocarcinoma cell lines were obtained from the Cell Bank of the Chinese Academy of Sciences (Shanghai, China). All these cells were cultured in RPMI 1640 medium with additional 10% fetal bovine serum (Gibco, Carlsbad, CA, USA) and 1% penicillin–streptomycin solution (Beyotime, Shanghai, China). Moreover, cobalt chloride (CoCl_2_) (232696, Sigma-Aldrich, St. Louis, MO) was used to mimic hypoxic conditions. Under some conditions, cells were treated with 150 μM CoCl_2_ for 48 h and then subjected to RNA or protein extraction.

### Establishment of stable cell lines

The methods used to construct stable NRP1 knockdown and overexpression cell lines were reported in our previous study^19^. To construct a stable HIF-1α knockdown cell line, two DNA fragments (HIF-1α shRNA-1, 5′-AGUUCACCUGAGCCUAAUATT-3U or HIF-1α shRNA-2, 5′-CAAGCAACUGUCAUAUAUATT-3′AG) were subcloned into the lentiviral vector pGMLV-SC5 (Genomeditech, Shanghai, China) by digestion with the endonucleases BamHI and EcoRI. To generate HIF-1α-overexpressing cells, a 2481-bp fragment of the HIF-1α coding sequence was synthesized (Genewiz, Suzhou, China), and subcloned into the PLVX-IRES-Neo vector using the endonucleases SwaI and NotI. HIF-1α-silenced or HIF-1α-overexpressing constructs or controls were cotransfected with packaging plasmids into human embryonic kidney 293 cells using Lipofectamine 2000 (Invitrogen, San Diego, CA, USA). Forty-eight hours later, we collected the supernatant to infect A549 and SPC-A1 cells at least three times and then selected positive cells with 0.4 µg/ml puromycin (Sigma-Aldrich, St. Louis, MO).

### RNA extraction and quantitative real-time polymerase reaction (qRT-PCR)

All procedures were performed as previously described^21^. The following primers were used for qRT-PCR: for HIF-1α, 5′-ATCCATCTGACCATGAGGAAATG-3′ (forward) and 5′-TCGGCTAGTTAGGGTACACTTC-3′ (reverse); and for NRP1, 5′-GAAAAATGCGAAT

GG CTGAT-3′ (forward) and 5′-AATGGCCCTGAAGACACAAC-3′ (reverse). β-actin was used as internal control. For β-actin, the primer sequences were 5′-CACAGAGCCTCGCCTT

TGCC-3′ (forward) and 5′-ACCCATGCCCACCATCACG-3′ (reverse). Relative fold changes were calculated using the 2^−ΔΔCt^ method.

### Western blot analysis

All procedures were performed as we previously reported^22^. The antibodies used in our current study were anti-NRP1 (A-12) (Santa Cruz Biotechnology, CA, USA), anti-HIF-1α (D1S7W), anti-VE-cadherin (D87F2), anti-MMP2 (D8N9Y) (Cell Signaling Technology, Danvers, MA, USA), and anti-Vimentin (RV202) (BD Biosciences, Oxford, UK). Anti-β-actin and anti-mouse or anti-rabbit secondary antibodies from Cell Signaling Technology were used.

### Transwell assay

A549 and SPC-A1 lung adenocarcinoma cells (25 × 10^4^ cells/ml) were suspended in RPMI 1640 medium with 1% FBS. For the migration assay, 200 µl of the cell suspension was added to the upper transwell inserts. For the invasion assay, all inserts were precoated with Matrigel matrix (BD Biosciences, Sparks, MD) diluted in 0% medium and then incubated at 37 °C for 2 h. Then, 200 µl medium was added to the upper precoated inserts. All lower chambers were filled with 800 µl medium containing 10% FBS. Twenty-four hours later, the inserts were fixed with methanol and stained with 0.5% crystal violet. Images were acquired under a microscope (IX73, Olympus).

### Wound healing assay

For wound-healing assays, A549 and SPC-A1 lung adenocarcinoma cells were plated in 6-well culture plates. After monolayer formation, a straight scratch was made across the center of the well using a 10 μl pipette tip. The cells were gently washed with PBS and then replenished with serum-free medium. The cells were cultured for an additional 24 h, and images were acquired under a microscope (IX73, Olympus).

### Three-dimensional culture

To monitor VM formation, 50 μl thawed Matrigel matrix (BD Biosciences, Sparks, MD) was added to 96-well plates and then kept at 37 °C for 2 h. A549 and SPC-A1 cells after the indicated treatments were suspended in complete medium at a density of 5–10 × 10^5^ cells/ml. Two hours later, 100 μl of cell suspension was seeded onto the gel in each well and cultured for 24 h. The formation of VM structures was observed and captured under a microscope (IX73, Olympus) at ×100 magnification.

### Immunofluorescence staining

Cells were seeded in 12-well plates that were preinserted with glass slides. Twenty-four hours later, when the cells reached 40–50% confluence, they were washed with PBS. The cells were then fixed with 4% paraformaldehyde for 30 min followed by permeabilization with 0.5% Triton X-100 solution for an additional 20 min. Next, 5% bovine serum albumin was added to function as the blocking buffer. The primary antibodies used in our experiment were anti-HIF-1α (D1S7W) and anti-NRP1 (A-12). The corresponding secondary antibodies tagged with Cy3 and FITC were used (1:500, Beyotime Biotechnology).

### Plasmid construction, transient transfection, and luciferase assay

A 2000-bp fragment of the NRP1 promoter containing potential HIF-1α binding sites was fused to the 3′-end of a luciferase reporter for the luciferase assay. Plasmids containing wild-type (WT) NRP1 promoter (pGL3-NRP1) or mutated fragments of the NRP1 promoter (pGL3-NRP1-mutant1/mutant2/mutant1 and 2) were directly synthesized (Genewiz, Suzhou, China). A549 and SPC-A1 cells were plated in a 24-well plate and cotransfected with the constructed plasmids (500 ng) together with a Renilla pRL-TK plasmid (40 ng) using Lipofectamine 2000 (Life Technologies, Carlsbad, CA). Twenty-four to forty-eight hours later, the cell lysates were collected, and luciferase activity was examined with a Dual-Luciferase Reporter Assay Kit (Promega).

### Chromatin immunoprecipitation (ChIP) assay

A ChIP-IT Kit (Active Motif, Carlsbad, CA, USA) was used to perform the ChIP assay. Briefly, cells were fixed with formaldehyde and then lysed. To precipitate the DNA fragment, 2 μg anti-HIF-1α antibody or normal IgG was used. The DNA-protein complexes were pulled down with magnetic beads and then de-crosslinked. The extracted DNA samples were then amplified with NRP1 promoter-specific primers.

### Statistical analysis

All experiments were independently performed in triplicate as a minimum. All statistical analyses were performed using GraphPad Prism 7.0 (GraphPad, San Diego, CA, USA) and SPSS 17.0 software (SPSS, Chicago, IL, USA). All data were presented as the mean ± SD. Significant differences between two groups were assessed by a nonpaired Student’s *t*-test. Significant differences between three or more groups were analyzed using one-way or two-way ANOVA followed by the Bonferroni post hoc test. All statistical tests were two-tailed. *p* < 0.05 was set as statistically significant.

## Results

### The correlations between HIF-1α or NRP1 expression as well as VM and the clinicopathological parameters and prognosis of patients with LUAD

**Fig. 1:**
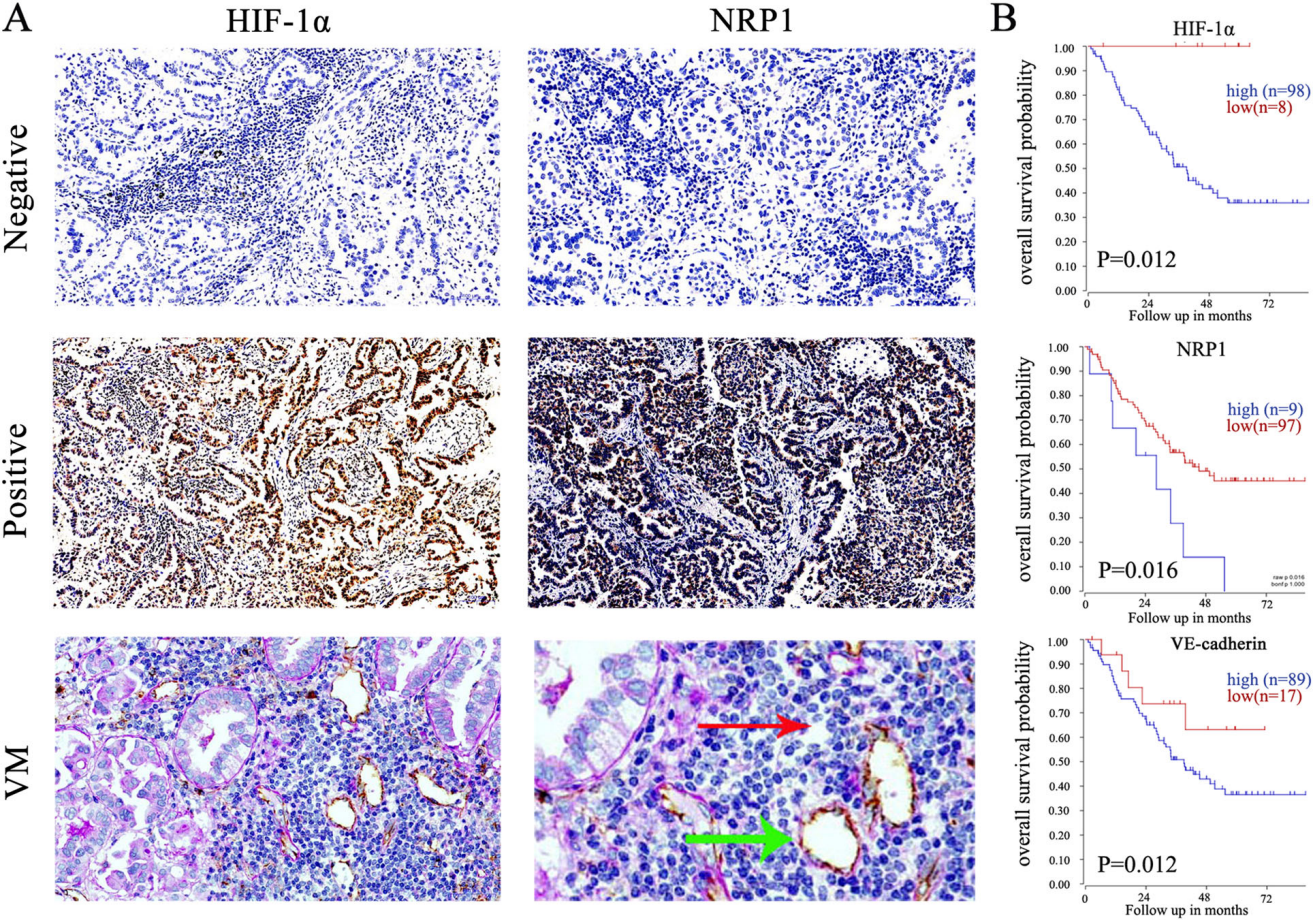
The correlation between HIF-1α or NRP1 expression as well as VM and the clinicopathological parameters and prognosis of patients with LUAD. **A** LUAD specimens were analyzed by immunohistochemistry. The upper panel shows negative expression, and the middle panel shows positive expression of HIF-1α and NRP1. The lower panel represents VM (×200, scale bars, 100 µm). The red arrows indicate VM channels, which are characterized by PAS^+^CD34^−^ tumor cells, and the green arrows indicate EDVs, which are characterized by PAS^+^CD34^+^ tumor cells. **B**, **C** Kaplan–Meier analysis of the overall survival of patients with HIF-1α-high and HIF-1α-low samples, NRP1-high and NRP1-low samples, and VE-cadherin-high and VE-cadherin-low samples.

We detected the HIF-1α and NRP1 expression as well as the presence of VM in 169 LUAD specimens. IHC assays showed that HIF-1α protein was overexpressed in 88 of the 169 LUAD sample tissues (52.07%). NRP1 was highly expressed in 86 of the 169 LUAD sample tissues (50.88%) (Fig. 1A and Table 1). Tubes characterized by PAS^+^CD34^−^ tumor cells within the cavities were considered VM channels (red arrows). Both PAS and CD34 positive channels were defined as EDVs (green arrows, Fig. 1A). VM was detected in 32 of the 169 LUAD tissues (18.93%; Fig. 1A). Further clinically relevant analysis showed that elevated HIF-1α and NRP1 expression was associated with lymph node metastasis. More interestingly, we found that HIF-1α and NRP1 expression were significantly correlated with the presence of VM (*p* < 0.05, Table 1). Moreover, the presence of VM was positively correlated with TNM grade and lymph node metastasis (*p* < 0.05, Table 2). As expected, a positive correlation between NRP1 and HIF-1α expression was found in 169 LUAD sample tissues (Fig. S1). Moreover, we confirmed the positive relationship between NRP1, HIF-1α, and VE-cadherin expression in lung cancer tissues via the public GEPIA, UCSC, and CCLE databases (Fig. S2). Finally, Kaplan–Meier survival analysis indicated that high HIF-1α, NRP1, or VE-cadherin expression indicated poor overall survival (*p* < 0.05, Fig. 1B).

**Table 1:** Relationship between HIF-1α, NRP1 and the clinicopathological features in Lung adenocarcinoma.

Variants	HIF-1α		*χ*^2^	*p* value	NRP1		*χ*^2^	*p* value
	−	+			−	+		
*Sex*								
Male	40	53	2.004	0.157	43	50	0.684	0.408
Female	41	35			40	36		
*Age (years)*								
<65	46	62	3.414	0.065	53	55	0	0.989
≥65	35	26			30	31		
*Tumor size (cm)*								
<5	60	59	1	0.317	64	55	3.509	0.061
≥5	21	29			19	31		
*Grade*								
I + II	57	57	0.602	0.438	56	58	0.03	0.864
III + IV	24	31			27	28		
*Metastasis*								
No	66	58	5.235	0.022	67	57	5.173	0.025
Yes	15	30			16	29		
*VM*								
No	72	65	6.203	0.013	73	64	4.315	0.038
Yes	9	23			10	22		

*P* < 0.05 was statistically significant.

**Table 2:** The correlation of VM with the clinicopathological parameters of lung adenocarcinoma.

Variants	VM			
	Negative	Positive	*χ*^2^	*p*-value
*Sex*				
Male	76	17	0.058	0.81
Female	61	15		
*Age (years)*				
<65	91	18	1.172	0.279
≥65	46	14		
*Tumor size (cm)*				
<5	99	20	3.242	0.072
≥5	28	12		
*Grade*				
I + II	101	19	5.607	0.018
III + IV	36	13		
*Metastasis*				
No	108	12	21.526	0.000
Yes	29	20		

*P* < 0.05 was statistically significant.

### HIF-1α induced NRP1 expression in lung adenocarcinoma cells

**Fig. 2:**
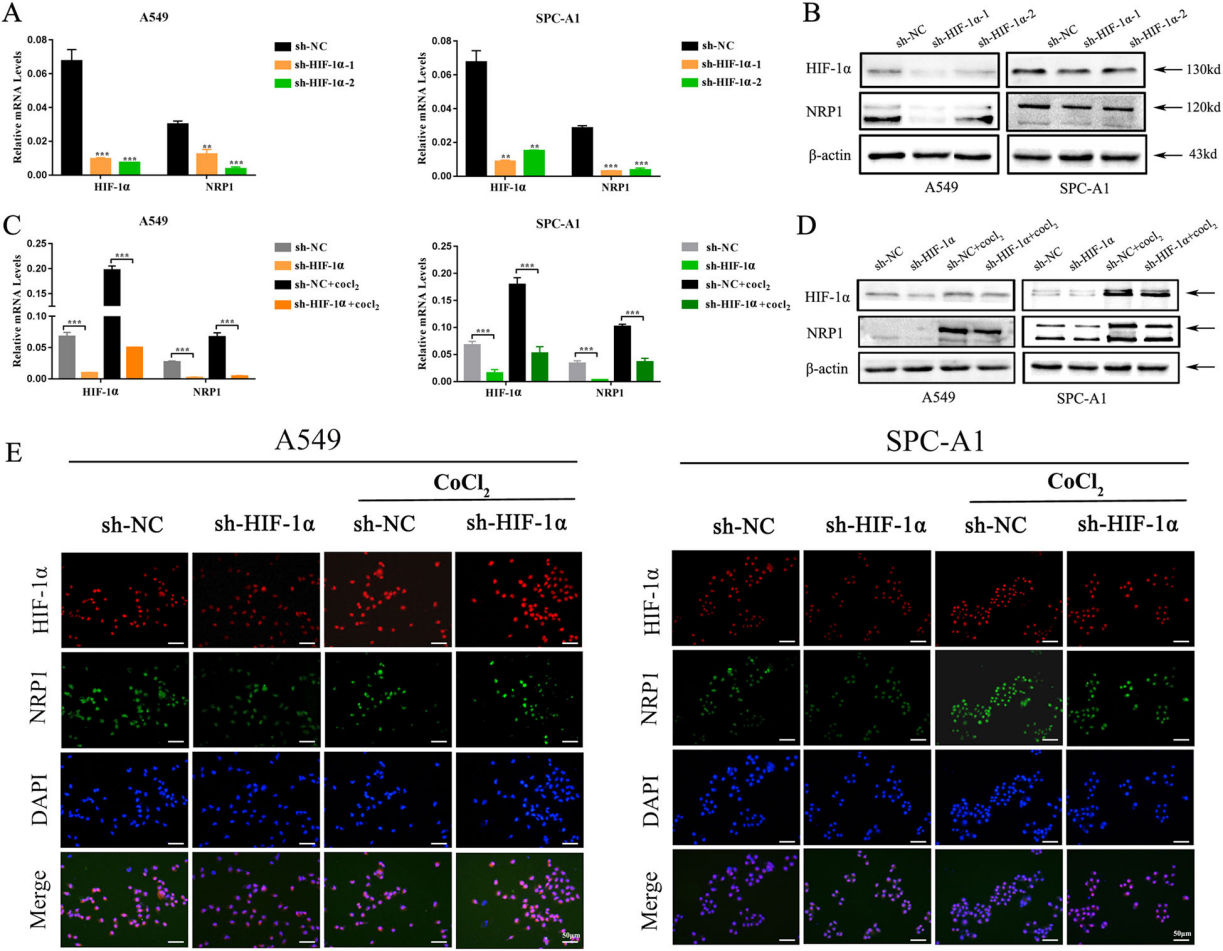
HIF-1α induced NRP1 expression in lung adenocarcinoma cells. **A**, **B** The mRNA and protein expression levels of HIF-1α and NRP1 in A549 and SPC-A1 cell lines after stable knockdown of HIF-1α expression were assessed via qRT-PCR and western blot assays, respectively. **C**, **D** The stable HIF-1α knockdown cell lines and the control cells were treated with 150 μM CoCl_2_ for 48 h. HIF-1α and NRP1 expression were detected at both the mRNA and protein levels. **E** Immunofluorescence staining in stable HIF-1α knockdown cell lines treated with or without CoCl_2_ (scale bar, 50 µm). *p < 0.05.

To verify the role of HIF-1α in regulating NRP1 expression, HIF-1α expression was detected in NSCLC cell lines. We found that HIF-1α expression was upregulated in NSCLC cell lines compared to BEAS-2B cell lines at both the mRNA and protein levels (Fig. S3). Then, we stably knocked down HIF-1α in A549 and SPC-A1 cell lines with two individual short hairpin RNAs (sh-HIF-1α). Downregulated HIF-1α expression was confirmed via qRT-PCR and western blot assays. Interestingly, we found that NRP1 expression was downregulated at both the mRNA and protein levels after HIF-1α knockdown (Fig. 2A, B). To confirm that HIF-1α induces NRP1 expression in lung adenocarcinoma cells, A549 and SPC-A1 cells with stable knockdown of HIF-1α were treated with the hypoxia-mimetic agent CoCl_2_ (150 μM) for 48 h. The data showed that CoCl_2_ induced HIF-1α expression, and HIF-1α knockdown inhibited the CoCl_2_-induced upregulation of NRP1 (Fig. 2C, D). Furthermore, immunofluorescence assays revealed similar results (Fig. 2E). Collectively, all these results demonstrated that NRP1 expression was induced by HIF-1α in the hypoxic tumor microenvironment.

### NRP1 overexpression enhanced the aggressive phenotype under HIF-1α knockdown

**Fig. 3:**
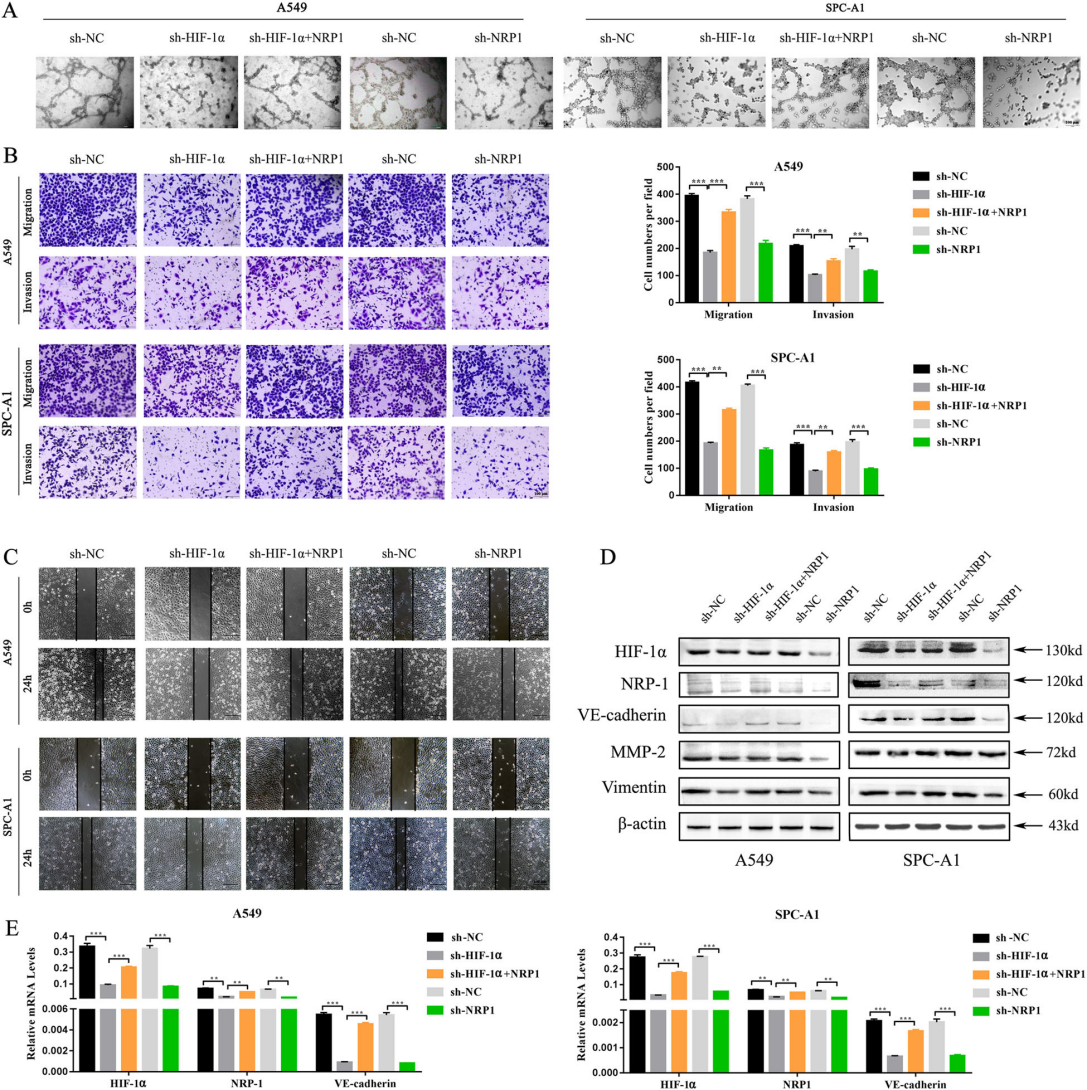
NRP1 overexpression enhanced the aggressive phenotype under HIF-1α knockdown. **A** The effects of HIF-1α and NRP1 knockdown on the tube formation abilities of the A549 and SPC-A1 cell lines. **B** Transwell assay of the invasion and migration ability of LUAD cells after NRP1 or HIF-1α knockdown followed by NRP1 overexpression (**B** ×40; scale bars, 100 μm). **C** Wound healing assay of the migratory ability of LUAD cells after NRP1 or HIF-1α knockdown followed by NRP1 overexpression (**B**, ×40; scale bars, 100 μm). **D** Western blot assay of HIF-1α, NRP1, VE-cadherin, MMP-2, Vimentin, and β-actin expression levels. **E** The mRNA expression levels of HIF-1α, NRP1, and VE-cadherin in the A549 and SPC-A1 cell lines after NRP1 or HIF-1α knockdown followed by NRP1 overexpression. *p < 0.05.

To assess whether the effects of HIF-1α on LUAD cells are mediated by NRP1 expression, rescue assays were performed. First, we evaluated the role of HIF-1α/NRP1 in mediating the aggressive phenotypes. Tube formation assays showed that NRP1 or HIF-1α knockdown alone inhibited VM formation, whereas NRP1 overexpression facilitated the development of VM tubes after HIF-1α expression was silenced (Fig. 3A). Moreover, the transwell assay and wound-healing assay showed that the numbers of migrated cells were decreased under HIF-1α or NRP1 knockdown conditions. Moreover, restoring NRP1 expression greatly reduced the inhibitory influence of HIF-1α knockdown on cell migration and invasion (Fig. 3B, C). Hypoxia, aberrant extravascular expression of VE-cadherin and EMT were all regarded as significant factors in VM formation and tumor metastasis^23,24^. Thus, we evaluated VE-cadherin and EMT-related marker expression using western blot and qRT-PCR assays. We found that VE-cadherin, MMP2, and Vimentin expression were downregulated following HIF-1α or NRP1 knockdown. Conversely, cells overexpressing NRP1 demonstrated upregulated VE-cadherin, MMP2, and Vimentin expression compared to the sh-HIF-1α group cells (Fig. 3D, E).

### NRP1 knockdown counteracted the aggressive phenotype induced by HIF-1α

**Fig. 4:**
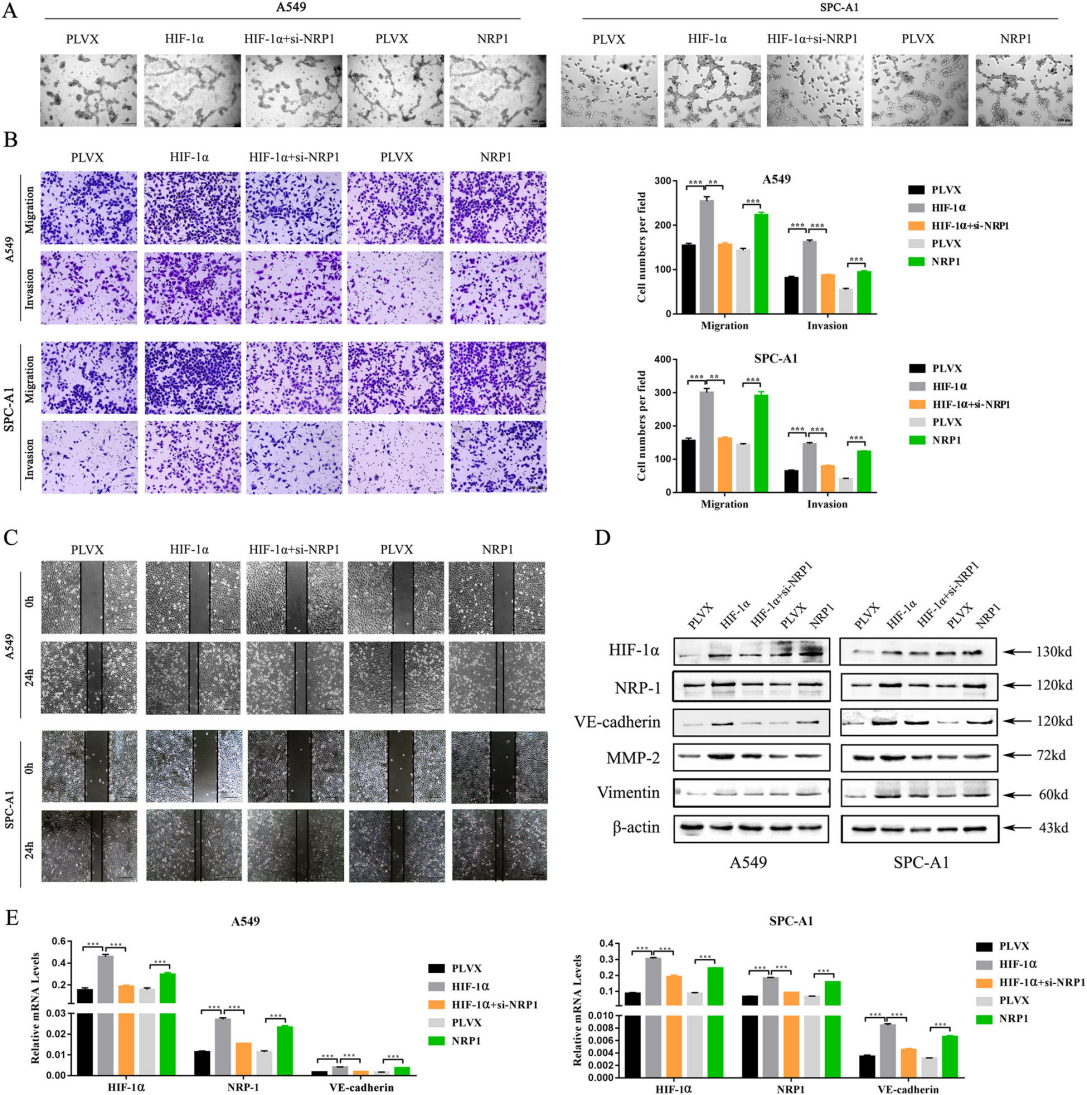
NRP1 knockdown counteracted the aggressive phenotype induced by HIF-1α. **A** Effects of HIF-1α and NRP1 overexpression on the tube formation abilities of the A549 and SPC-A1 cell lines. **B** Transwell assay of the invasion and migration ability of LUAD cells after NRP1 or HIF-1α overexpression followed by NRP1 knockdown (**B**, ×40; scale bars, 100 μm). **C** Wound healing assay of the migratory ability of LUAD cells after NRP1 or HIF-1α overexpression followed by NRP1 knockdown (**B**, ×40; scale bars, 100 μm). **D** Western blot assay of HIF-1α, NRP1, VE-cadherin, MMP-2, Vimentin, and β-actin expression levels. **E** The mRNA expression levels of HIF-1α, NRP1, and VE-cadherin in the A549 and SPC-A1 cell lines after NRP1 or HIF-1α overexpression followed by NRP1 knockdown. *p < 0.05.

Next, we confirmed the above findings in HIF-1α-overexpressing cell lines. Tube formation assays showed that overexpression of either HIF-1α or NRP1 resulted in the formation of tube-like structures. However, silencing NRP1 triggered the disappearance of these VM structures even if HIF-1α was overexpressed (Fig. 4A). The transwell assay and wound-healing assay showed that the numbers of migrated cells were increased after HIF-1α or NRP1 overexpression. However, NRP1 knockdown counteracted the increased migratory and invasive abilities of LUAD cells that were induced by HIF-1α (Fig. 4B, C). As expected, the elevated VE-cadherin, MMP2, and Vimentin expression induced by HIF-1α was downregulated following NRP1 knockdown (Fig. 4D, E).

### NRP1 is a direct target of HIF-1α

**Fig. 5:**
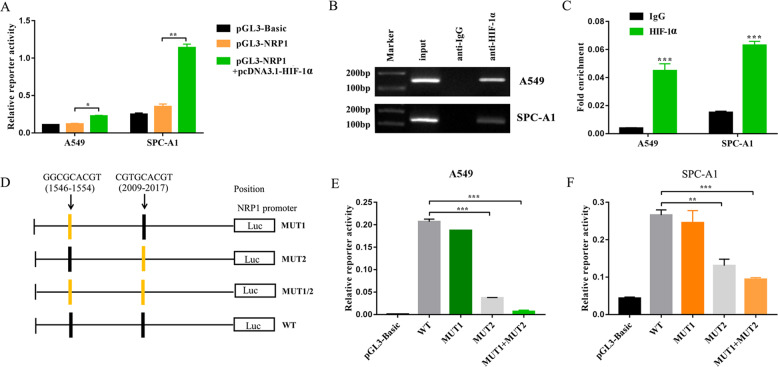
NRP1 is a direct target of HIF-1α. **A** Luciferase reporter assay in A549 and SPC-A1 cells after transfection with pGL3-reporter plasmids containing the NRP1 promoter or basic vector with or without pcDNA3.1-HIF-1α for 24 h. **B**, **C** ChIP assay was performed to detect whether HIF-1α binds to the NRP1 promoter in A549 and SPC-A1 cells. **D** Diagram of the NRP1 promoter structure with potential or mutant HIF-1α binding sites. **E** Luciferase reporter assay in A549 and SPC-A1 cells after transfection with a set of luciferase reporter plasmids containing potential binding sites of the NRP1 promoter or mutants at two HIF-1α binding sites for 24 h.

Previous observations demonstrated that HIF-1α regulates NRP1 expression at both the mRNA and protein levels, and thus promotes NRP1-induced VM and migration. Next, we further explored how HIF-1α regulates NRP1 expression. HIF-1α is well known for its role in transcriptional activity through direct or indirect mechanisms^25,26^. To determine whether NRP1 is a direct target of HIF-1α, a 759-bp fragment of the NRP1 promoter was subcloned into the pGL3 vector to construct the pGL3-NRP1 plasmid. Luciferase assays showed that HIF-1α overexpression activated NRP1 promoter activity in both the A549 and SPC-A1 cell lines (Fig. 5A). ChIP assay was also performed, and DNA fragments bound by endogenous HIF-1α were immunoprecipitated. We found significant enrichment of HIF-1α at the NRP1 promoter (Fig. 5B, C). Then, the NRP1 promoter was searched for potential hypoxia-response elements (HREs). Sequence analysis indicated that two consensus HRE sites were located in the regions from −1546 to −1554 and −2009 to −2017 (Fig. 5D). Mutation of the second or both HIF-1α binding sites abrogated luciferase activity, suggesting that HIF-1α directly binds to the NRP1 promoter and activates NRP1 expression (Fig. 5E, F).

## Discussion

Two models of blood vessel formation in malignant tumors have been reported. In addition to traditional tumor angiogenesis and vasculogenesis, VM, formed by highly aggressive cancer cells, has been accepted as a new model of neovascularization to provide sufficient blood supply for tumor growth^27,28^. Tumor stem cells possess the capacity for self-renewal and differentiation, which accounts for tumor vascularization^29^. VM has been found in many malignant tumors, including NSCLC. In addition, high expression of VM indicated poor prognosis, low survival, and aggressive invasion and metastasis in cancer patients, suggesting that it was a novel hallmark of cancer^30^.

Hypoxia is a distinguishing feature in most solid tumors. Recently, a growing body of evidence has shown that the hypoxic microenvironment can regulate various signaling pathways involved in cellular differentiation, maintenance of stem cell characteristics, tumor progression, angiogenesis, and VM^31^. Hypoxia-induced overexpression of HIF-1α is associated with VM in many cancer types^10^. In addition, HIF-1a overexpression can activate genes involved in tumor development and aggression, and thus is regarded as an unfavorable prognosis in cancers^32^. Here, we showed that HIF-1α is highly expressed in LUAD patient specimens. Furthermore, HIF-1α accumulation is associated with VM and poor patient survival. In vitro studies also showed that HIF-1α knockdown inhibited LUAD cell migration, invasion, and VM. However, how HIF-1α regulates cell migration and VM formation has remained highly debated.

NRP1 is a transmembrane cell surface coreceptor that serves as a receptor for the VEGF_165_ isoform of vascular endothelial growth factor (VEGF) to enhance angiogenesis, vascular permeability, and arteriogenesis via interaction with VEGF receptor 2 (VEGFR2)^33^. In addition, NRP1 regulates angiogenesis by activating the intracellular kinase ABL1 independent of VEGF signaling^34^. NRP1 also acts as a receptor for the class 3 semaphorin (SEMA3A) to regulate vascular permeability and vessel maturation during tumor angiogenesis^13^. However, until now, limited studies have reported the role of NRP1 in promoting VM formation. In the current study, we found that NRP1 overexpression is associated with VM and poor patient survival in LUAD patient specimens. We proved that NRP1 affects the migration and invasion of LUAD cells, which was in line with our previously published results. In addition, we also found that blocking NRP1 inhibits VM formation, while NRP1 overexpression promoted VM formation.

HIF-1α plays a central role in bone marrow-derived EPC (bmEPC) homing and sprouting in the postacute stage of ischemic Sprague–Dawley (SD) rat brains by regulating NRP1 expression^35^. HIF-1α was also reported to promote glycolysis in pancreatic cancer by regulating NRP1^36^. However, whether the HIF-1α/NRP1 axis functions in VM formation has not been reported. ChIP assay showed that NRP1 is a direct target of HIF-1α. Migration and invasion assays, wound healing assays, and VM formation assays showed that NRP1 knockdown counteracted the aggressive phenotype induced by HIF-1α, while NRP1 overexpression enhanced the aggressive phenotype under HIF-1α knockdown. All data suggested that the HIF-1α/NRP1 axis regulated LUAD cell migration and VM formation.

Furthermore, we deeply explored the underlying mechanisms of HIF-1α/NRP1 axis-mediated cell migration and VM formation. It has been reported that high levels of matrix metalloproteinases, such as MMP-1, MMP-2, MMP-9, and MMP-14, produced by tumor cells participate in VM^37^. A hypoxic tumor microenvironment promotes HIF1-α-induced EMT and thus enhances the invasive and migratory abilities and VM in tumors. Vascular endothelial (VE)-cadherin is a major endothelial adhesion molecule that controls cell–cell junctions and blood vessel formation in tumor development^38^. It has been reported that HIF-1α binds to the promoter of VE-cadherin and promotes its expression at the transcriptional level^39^. In addition, VE-cadherin promotes vasculogenic mimicry by modulating the expression of kaiso-dependent genes, such as WNT11 and CCDN1^23^. Therefore, we next verified whether the HIF-1α/NRP1 axis activated MMPs and VE-cadherin in LUAD. Interestingly, we found that the elevated VE-cadherin, MMP2, and Vimentin expression induced by HIF-1α was downregulated following NRP1 knockdown.

**Fig. 6:**
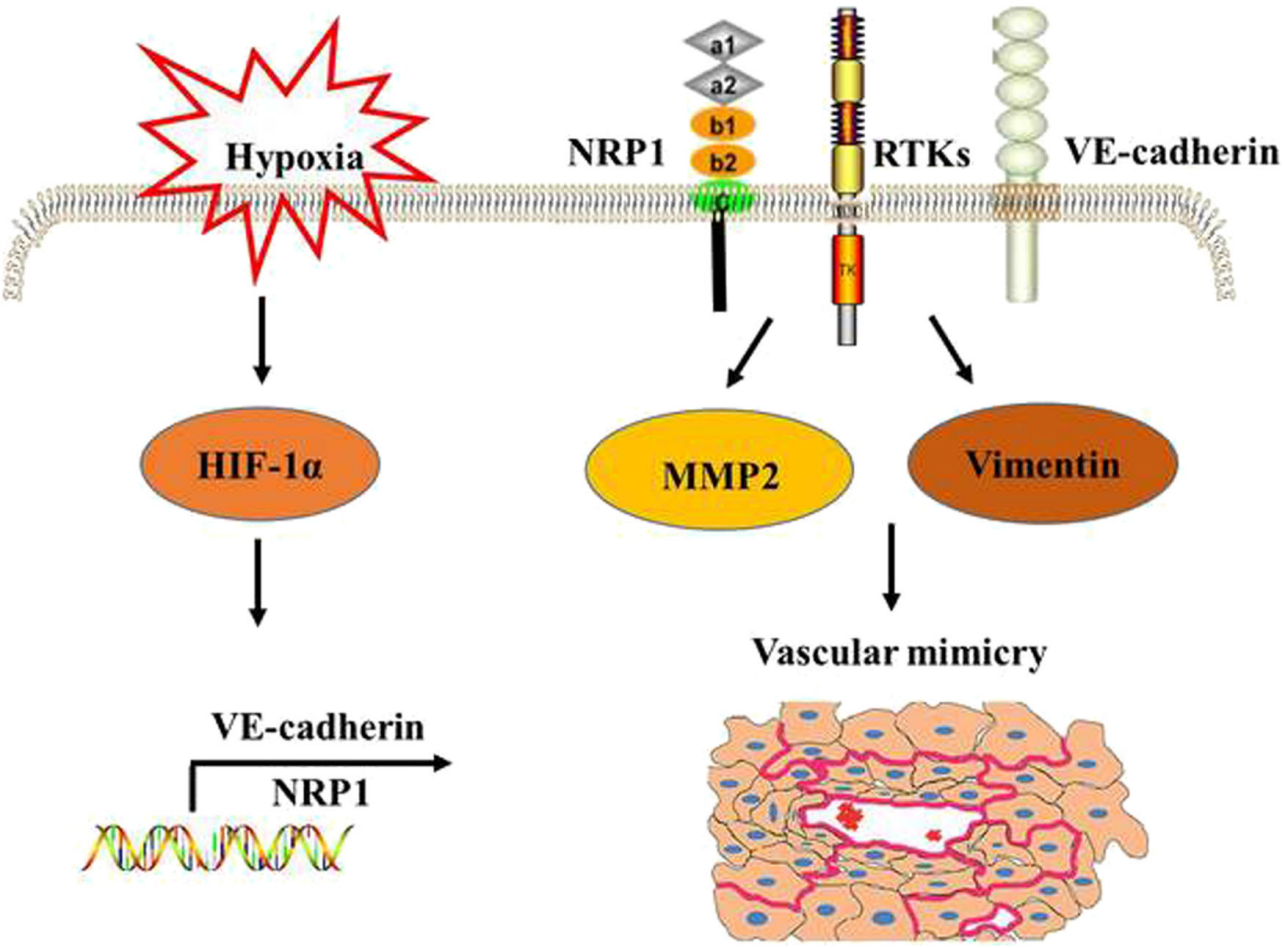
The proposed mechanistic model underlying HIF-1α/NRP1-mediated VM in LUAD. In tumor microenvironment, HIF-1α/NRP1 signaling can subsequently result in activation of MMP2, Vimentin, and VE-cadherin and thus promote EMT and VM, which ultimately contribute to tumor progression in LUAD.

Here, we demonstrated a new regulatory axis in the hypoxic tumor microenvironment involving elevated NRP1 expression induced by HIF-1α. HIF-1α/NRP1 signaling can subsequently result in activation of MMP2, Vimentin, and VE-cadherin and thus promote EMT and VM, which ultimately contribute to tumor progression in LUAD (Fig. 6).

## Supplementary information

Figure S1

Figure S2

Figure S3

Supplementary figure legends
