# Supplementary figures and images for: HIF-1α promoted vasculogenic mimicry formation in lung adenocarcinoma through NRP1 upregulation in the hypoxic tumor microenvironment
Source: Cell Death Dis. 2021 Apr 13;12(4):394. doi: 10.1038/s41419-021-03682-z (PMC8044151; doi:10.1038/s41419-021-03682-z)

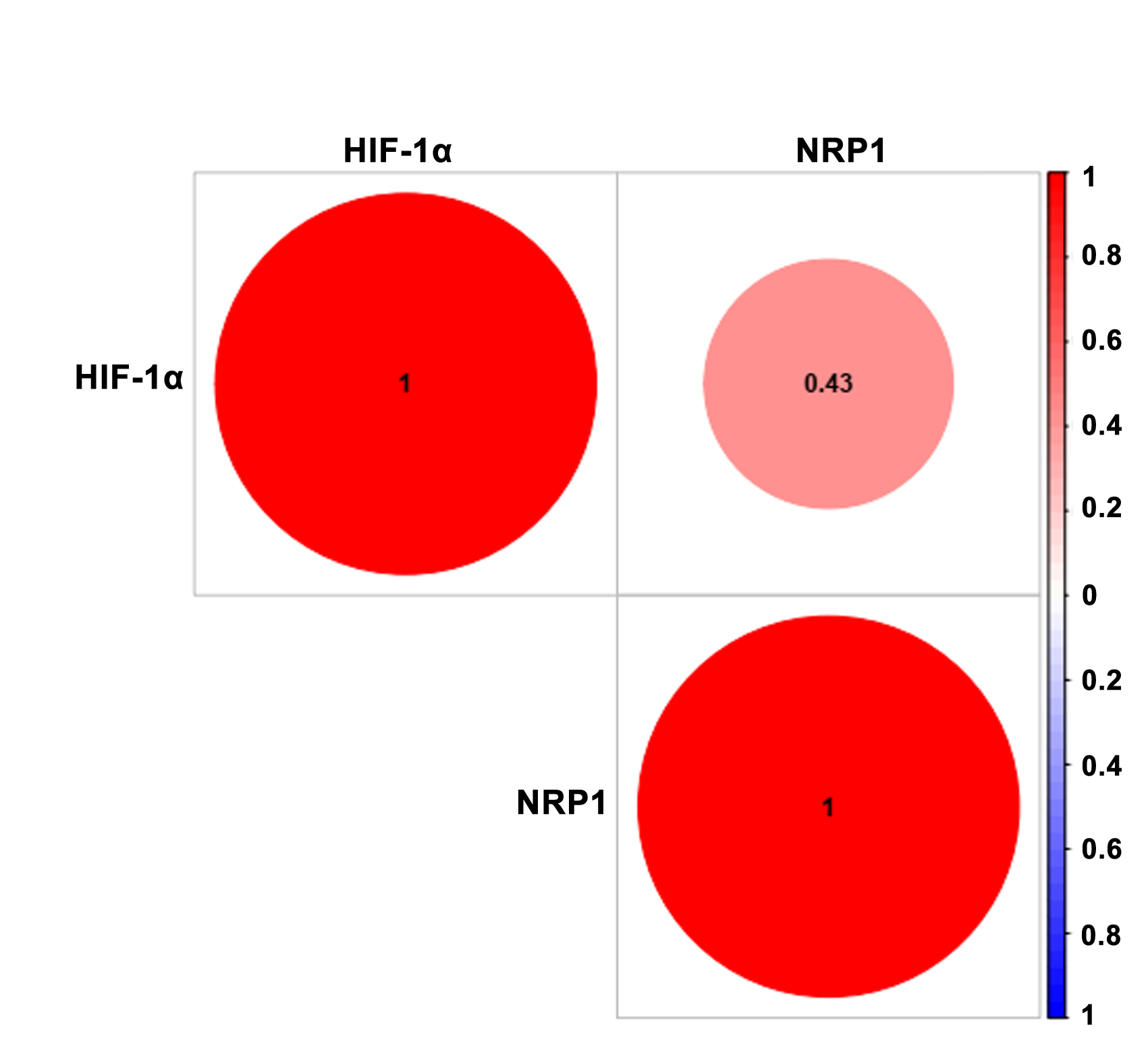

Supplement: Supplementary file 1 — Figure S1 [file 41419_2021_3682_MOESM1_ESM.tif]

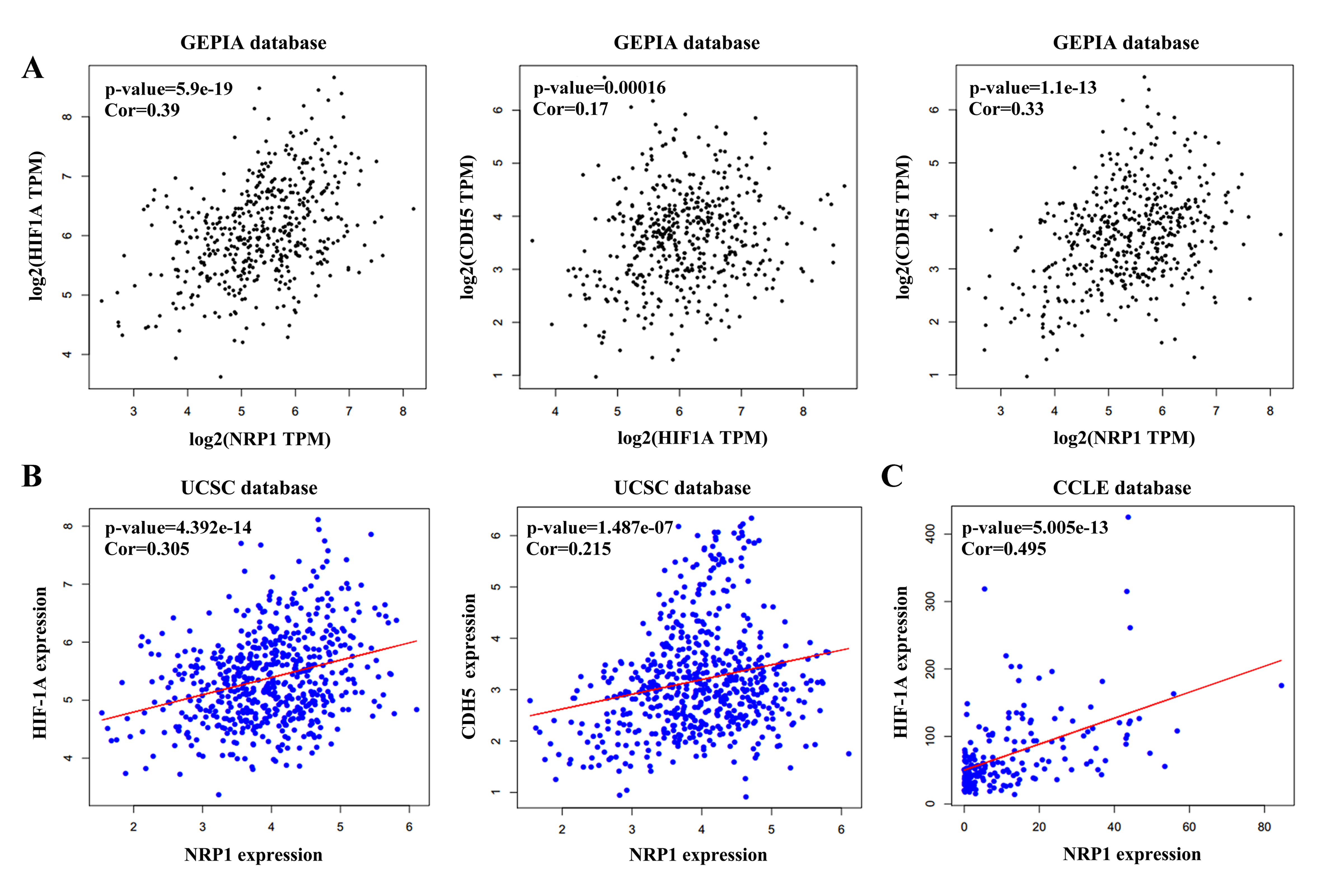

Supplement: Supplementary file 2 — Figure S2 [file 41419_2021_3682_MOESM2_ESM.tif]

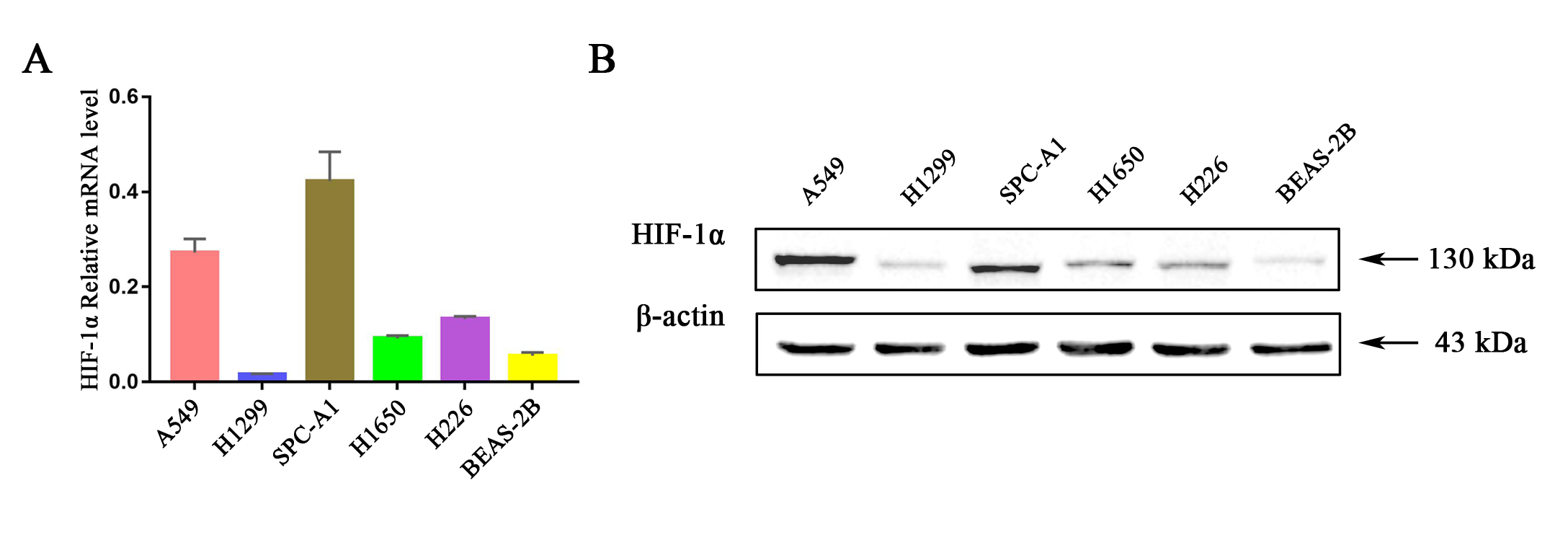

Supplement: Supplementary file 3 — Figure S3 [file 41419_2021_3682_MOESM3_ESM.tif]
