# Supplementary material for: HIF-1α promoted vasculogenic mimicry formation in lung adenocarcinoma through NRP1 upregulation in the hypoxic tumor microenvironment
Source: Cell Death Dis. 2021 Apr 13;12(4):394. doi: 10.1038/s41419-021-03682-z (PMC8044151; doi:10.1038/s41419-021-03682-z)
Supplement: Supplementary file 4 — Supplementary figure legends [file 41419_2021_3682_MOESM4_ESM.docx]

**Supplementary figures**

**Fig. S1. The correlation between HIF-1α and NRP1 in 169 LUAD sample tissues.**

Positive correlation was found between HIF-1α and NRP1 expression in our involved LUAD tissues.

**Fig. S2. The correlations among HIF-1α, NRP1, and VE-cadherin in lung cancer tissues via public databases.**

a Positive correlations were found among HIF-1α, NRP1, and VE-cadherin expression via data from the GEPIA database. b Data from the UCSC database showed a positive relationship between NRP1 and HIF-1α expression. c A positive correlation between NRP1 and HIF-1α or VE-cadherin was confirmed via data from the CCLE database.

**Fig. S3. The expression of HIF-1α in NSCLC cell lines.**

**a, b** qRT-PCR and western blot analysis of HIF-1α expression in the A549, H1299, SPC-A1, H1650, and H226 cell lines compared to that in the BEAS-2B cell line.
